# Novel Treatment Concepts for Cervical Cancer—Moving Towards Personalized Therapy

**DOI:** 10.3390/jpm15110523

**Published:** 2025-11-01

**Authors:** Melina Danisch, Magdalena Postl, Thomas Bartl, Christoph Grimm, Alina Sturdza, Nicole Concin, Stephan Polterauer

**Affiliations:** 1 Department of Obstetrics and Gynecology, Comprehensive Cancer Center Vienna, Division of General Gynecology and Gynecological Oncology, Medical University of Vienna, 1090 Vienna, Austria; melina.danisch@meduniwien.ac.at (M.D.); magdalena.postl@meduniwien.ac.at (M.P.); thomas.bartl@meduniwien.ac.at (T.B.); nicole.concin@meduniwien.ac.at (N.C.); stephan.polterauer@meduniwien.ac.at (S.P.); 2 Department of Radiooncology, Medical University of Vienna, 1090 Vienna, Austria; alina.sturdza@meduniwien.ac.at

**Keywords:** cervical cancer, radical hysterectomy, sentinel lymph node dissection, immune checkpoint inhibitor, chemo-radiation, surgical treatment

## Abstract

In recent years, several randomized controlled trials have been published regarding cervical cancer therapy and significantly changed the treatment landscape. Recent advances have improved the treatment options and allow personalized treatment concepts with escalation of treatment in high-risk disease and de-escalation with reduction in morbidity in selected low-risk patients. This review aims to provide a comprehensive analysis of the latest landmark studies that are poised to significantly influence clinical practice. Personalized treatment concepts with careful patient selection allow de-escalation in the surgical treatment of cervical cancer. In low-risk cervical cancer patients (lesions of ≤2 cm with limited stromal invasion), simple hysterectomy (SH) was non-inferior to radical hysterectomy in terms of 3-year incidence of pelvic recurrence and was associated with a lower risk of urinary incontinence or retention and improved sexual health and quality of life. Furthermore, sentinel lymphadenectomy is constantly replacing systematic pelvic lymphadenectomy in patients with low-risk cervical cancer. In addition, further studies are necessary to clarify the role of postoperative therapy for patients with intermediate-risk cervical cancer. Starting in 2008, the EMBRACE studies assess the role of Image guided adaptive brachytherapy (IGABT) in LACC in addition to modern external beam radiotherapy concurrent to chemotherapy. The publication of the results of the EMBRACE I prospective study established MRI guided IGABT as state-of-the-art brachytherapy for LACC. EMBRACE II and additional prospective studies emerging from this consortium will address important questions in modern radiotherapy for LACC. Immune checkpoint inhibitors (CPIs) have been evaluated across various clinical settings and are expected to be utilized in numerous scenarios due to several positive randomized trials. Particularly, the combination of platinum-based chemotherapy and pembrolizumab, with or without bevacizumab, has been established as the new standard treatment for primary metastatic or recurrent PD-L1 positive high-risk cervical cancer. In locally advanced cervical cancer, two new treatment escalation regimens—neoadjuvant chemotherapy and adjuvant CPI therapy—have been evaluated in addition to chemoradiation. Furthermore, antibody-drug conjugates, such as tisotumab-vedotin, represent a promising future therapeutic option for recurrent cervical cancer.

## 1. Introduction

Cervical cancer ranks as the fourth most common malignancy among women worldwide [1]. In 2012, there were 528,000 new diagnoses and 266,000 deaths from cervical cancer globally. Eighty-five percent of these cases and deaths occur in low- and middle-income countries [2]. The highest incidence occurs between ages 40 and 59. Early detection through cytology smear tests has significantly reduced the incidence in countries like Germany and Austria [3]. Given the association of cervical cancer with HPV, there is an expectation that the implementation of HPV vaccination will further reduce incidence and mortality rates in the future [3].

Nevertheless, a substantial proportion of cervical cancers in Germany were diagnosed at advanced stages (23% at UICC stage III and 20% at stage IV) between 2013 and 2014 [4]. Management of advanced or recurrent stages has long been constrained by a lack of effective therapeutic strategies. Immune checkpoint inhibitors such as pembrolizumab are now a new therapeutic option, particularly as HPV-induced immunosuppression upregulates PD-L1, the target of pembrolizumab [5].

New studies show that de-escalation in the surgical treatment of cervical cancer does not worsen oncological outcomes, but reduces patient morbidity [6].

The aim of this narrative review is to present the latest advances in the treatment of cervical cancer. The most clinically relevant studies of recent years have been summarized here as an overview.

## 2. Early-Stage Cervical Cancer

In developed countries with high-quality radiotherapy, surgical procedures are mainly used in patients with early-stage cervical cancer (IA to IIA) without any risk factors. Traditionally early cervical cancer has been treated with radical hysterectomy (Wertheim) and systematic pelvic lymph node dissection (PLND) [3].

Recently several studies have been published supporting tailored treatment for low-risk cervical cancer and selective lymph node resection (Sentinel lymph node). These trials are summarized in Table 1.

### 2.1. SHAPE Trial

The multicenter, randomized SHAPE trial investigated whether radical hysterectomy provides a survival benefit over SH in patients with low-risk cervical cancer (lesions of ≤2 cm with limited stromal invasion) [6].

Seven hundred patients (350 in each group) were randomized, the majority of whom had stage IB1 tumours (FIGO of 2009, 91.7%).

The median duration of follow-up was 4.5 years. After 3 years, the rate of pelvic recurrence was 2.17% in the radical hysterectomy group compared to 2.52% in the SH group [6].

SH showed a lower incidence of urinary incontinence compared to the radical hysterectomy group (2.4% vs. 5.5%; p = 0.048), with similar results for the incidence of urinary retention (0.6% vs. 11.0%; p < 0.001) [6].

In conclusion, in a subgroup of early stage and low-risk cervical cancer patients, SH was non-inferior to radical hysterectomy in terms of 3-year incidence of pelvic recurrence. SH was linked to a decreased risk of urinary incontinence or retention [6].

A recent analysis found no evidence of worse clinical outcomes for minimally invasive surgery (MIS) compared to open surgery in patients who met the SHAPE criteria and underwent SH [7]. Nonetheless, because the surgical approach was not included in the randomization process, a large prospective study is required to confirm these results before routinely endorsing MIS for SH. The LASH trial will prospectively investigate the safety of MIS for early-stage cervical cancer [8].

### 2.2. LESSER Trial

This randomized phase-II trial investigated whether SH is inferior to modified radical hysterectomy (Querleu-Morrow type B2).

A total of 40 patients in stage IA2-IB1 (FIGO 2009) with tumours ≤2 cm at three oncological centers were included, who received either a SH (n = 20) or a modified radical hysterectomy (n = 20).

At a median follow-up of 52.1 months (IQR 43.9–60.1), the 3-year disease-free survival rate was 95% (95% CI 68% to 99%) after SH and 100% (95% CI 100% to 100%) after modified radical hysterectomy (log-rank p = 0.30) [9]. The respective 5-year overall survival (OS) rates were 90% (95% CI: 64–97%) and 91% (95% CI: 50–98%), with no significant difference between the groups (log-rank p = 0.46) [9]. No postoperative mortality was observed. Postoperative complication rates were comparable between the two groups (15% vs. 25%; p = 0.69), though the small sample size warrants cautious interpretation of these results.

The LESSER study suggests that SH could be a safe and potentially non-inferior alternative to radical surgery for patients with early-stage cervical cancer (≤2 cm) [9].

### 2.3. ConCerv Trial

The ConCerv trial, a prospective, single-arm, multicenter study, investigated the feasibility of conservative surgery in women with early-stage, low-risk cervical cancer [10].

The study included 100 patients diagnosed with stage IA2-IB1 cervical cancer (FIGO 2009), either squamous cell carcinoma (any grade) or adenocarcinoma (grade 1 or 2 only) [10]. Eligible patients had tumors smaller than 2 cm, no lymphatic space invasion, a depth of invasion less than 10 mm, negative imaging for metastatic disease, and negative conization margins. All participants had previously undergone cervical conization.

In patients opting for fertility-sparing treatment, a second intervention was performed to assess pelvic lymph nodes, involving either sentinel lymph node biopsy or full pelvic lymph node dissection [10]. Those who chose not to preserve fertility underwent an SH with lymph node assessment.

Lymph node assessment following conization was conducted in 44 women, while 40 women underwent conization followed by an SH with lymph node assessment. Additionally, 16 women underwent an accidental SH with subsequent lymph node dissection. Positive lymph nodes were identified in 5 patients (5%). Residual disease was detected in 1 out of 40 patients in the hysterectomy specimen after conization, indicating an immediate failure rate of 2.5% [10]. The median follow-up time was 36.3 months (range 0.0–68.3). Three patients experienced recurrence within two years post-surgery, with a cumulative incidence of 3.5% (95% CI: 0.9% to 9.0%) [10].

The ConCerv trial demonstrated that conservative surgery is a viable option for selected patients with early-stage, low-risk cervical cancer, as observed in this single-arm study [10].

### 2.4. LACC Trial

The randomized LACC trial investigated whether there is a difference in the survival rate of patients with early-stage cervical cancer who undergo either MIS or open abdominal radical hysterectomy.

Patients diagnosed with stage IA1 (with lymphatic invasion), IA2, or IB1 cervical cancer, along with a histological subtype of squamous cell carcinoma, adenocarcinoma, or adenosquamous carcinoma, were randomly allocated to undergo either MIS (319 patients) or open surgery (312 patients) [11].

At 4.5 years follow-up, disease-free survival was 86.0% for the MIS group and 96.5% for those who underwent open surgery, representing a difference of −10.6 percentage points (95% CI, −16.4 to −4.7) [11]. MIS was associated with lower DFS at 3 years (91% vs. 97%) and a higher risk of recurrence or death from cervical cancer (hazard ratio: 3.74; 95% CI, 1.63–8.58). Furthermore, overall survival at 3 years was also reduced in the MIS group (93.8% vs. 99.0%), with a hazard ratio for death from any cause of 6.0 (95% CI, 1.77–20.30).

These findings demonstrate that minimally invasive radical hysterectomy leads to inferior outcomes in terms of both DFS and OS compared to the open abdominal approach [11]. Of note, this trial had to be stopped earlier before completion of accrual due to the increased number of events in the endoscopic group.

### 2.5. SENTIX Trial

The multicenter prospective observational SENTIX trial assessed the use of SLN omitting systemic pelvic lymph node dissection in early-stage cervical cancer.

Within the SENTIX trial, SLN ultrastaging was performed in 647 of the 733 enrolled patients, revealing lymph node metastases in 13% of cases. The study population consisted of patients with cervical cancer stages ranging from IA1 with lymphovascular space invasion to IB2 (tumor size ≤4 cm, or ≤2 cm for those undergoing fertility-preserving treatment) who had no suspicious lymph nodes on preoperative imaging [12]. SLN assessment via frozen section and pathological ultrastaging was mandatory [12].

Patients were registered postoperatively if bilateral SLNs were detected in the pelvis and frozen section results were negative [12]. Bilateral SLNs were identified using one or a combination of the three main tracers: blue dye, radiocolloid, and indocyanine green.

Intraoperative assessment identified lymph node metastases in 57% of the affected patients (46 out of 81), with findings including macrometastases in 84%, micrometastases in 26%, and isolated tumor cells in 9%. SLN ultrastaging revealed additional metastatic disease in the remaining 43% (35 out of 81), with detailed sectioning detecting macrometastases or micrometastases at the first histological level in 25% (20 cases), between levels 2 and 4 in 11% (9 cases), and at level 5 or beyond in 7% (6 cases). Overall, ultrastaging identified 43% of additional node positive cases that were missed by imaging and intraoperative pathology.

The SENTIX trial, a single-arm study, demonstrated that high bilateral SLN detection is achievable using SLN biopsy in patients with tumors of 4 cm or less. In experienced centers, all SLNs were found in the pelvis, with most positioned below the bifurcation of the iliac vessels [12]. However, SLN assessment in frozen sections proved to be an unreliable intraoperative triage method, as it detected only about half of N1 cases. The detection rate of SLN metastatic involvement was correlated with the number of levels assessed through ultrastaging, with four levels recommended as the international standard for detecting over 90% of N1 cases [12].

### 2.6. SENTICOL Trial

In the present trial, a supplementary analysis of the two prospective multicenter studies Senticol I and II on SLN biopsy in patients with early-stage cervical cancer (FIGO stage IA to IIA) was performed.

The oncological outcomes of patients with negative lymph nodes who underwent either sentinel lymph node biopsy (SLNB) or pelvic lymphadenectomy (PL) were compared.

This trial included 259 patients, with 87 in the SLNB group and 172 in the PL group [13]. The median follow-up duration was 47 months (range: 4–127 months). Recurrences were observed in 21 patients (8.1%), including 4 cases of nodal recurrence (1.9%) [13]. A total of 9 patients (3.5%) died from cervical cancer. Disease-free survival (DFS) and disease-specific survival (DSS) were comparable between the SLNB and PL groups, with DFS rates of 85.1% vs. 80.4% (p = 0.24) and DSS rates of 90.8% vs. 97.2% (p = 0.22), respectively [13]. Multivariate Cox analysis showed no significant association between SLNB and either DFS (HR = 1.78, 95% CI: 0.71–4.46, p = 0.22) or DSS (HR = 3.02, 95% CI: 0.69–13.18, p = 0.14) when compared to PL [13]. The only independent predictor of DFS and DSS was the pathological risk level based on the Sedlis criteria.

The study results suggest that the omission of a complete pelvic lymphadenectomy in patients with bilateral negative SLN is not associated with an increased risk of recurrence [13].

### 2.7. SCCAN Trial

The following data analysis of the SCCAN database investigated the impact of the presence of macrometastases (MAC), micrometastases (MIC) and isolated tumour cells (ITC) in patients with cervical cancer at stage T1a1L1-T2b.

A total of 967 patient records from the SCCAN database were analyzed, all of whom had undergone primary surgery, including sentinel lymph node (SLN) biopsy and pathological ultrastaging.

In 172 patients (18%), lymph node metastases were identified and categorized into macrometastases (n = 79), micrometastases (n = 54), and isolated tumor cells (ITC; n = 39). Disease-free survival (DFS) was significantly reduced in patients with macrometastases (HR = 2.20, p = 0.003) and micrometastases (HR = 2.87, p < 0.001), with no meaningful difference observed between these two groups (p = 0.484) [14]. DFS in patients with ITC did not differ significantly from that in node-negative individuals (p = 0.127) or those with MAC/MIC involvement (p = 0.449).

A threshold analysis indicated that metastatic deposits ≥0.4 mm were associated with significantly decreased DFS compared to patients without nodal involvement (HR = 2.311, p = 0.04). However, due to limited statistical power, the prognostic impact of metastases <0.4 mm could not be reliably evaluated. No clear size threshold was found that conferred a survival advantage over other node-positive cases. LN metastases ≥0.4 mm showed a significant negative impact on DFS, while further subdivision of metastases based on their size showed no clinical significance [14].

A sub-analysis of the SCCAN study investigated whether adjuvant (chemo)radiation shows a survival benefit after radical surgery in patients with intermediate-risk early-stage cervical cancer.

A total of 692 patients who underwent primary surgery and met the following criteria were retrospectively analysed: Tumour size of 2–4 cm plus LVSI or a tumour size >4 cm; N0; no parametrial invasion; and clear surgical margins [15]. A total of 274 (39.6%) patients received no adjuvant treatment and 418 (60.4%) patients received radiotherapy or chemoradiotherapy.

No statistically significant differences were observed between the groups in terms of 5-year disease-free survival (83.2% vs. 80.3%; p = 0.365) or overall survival (88.7% vs. 89.0%; p = 0.281) [15]. Furthermore, the administration of adjuvant (chemo)radiotherapy was not associated with an improvement in survival outcomes.

In this study, radical surgery alone achieved comparable disease-free and overall survival rates to the combination of radical surgery and adjuvant (chemo-)radiotherapy [15].

### 2.8. Cervantes Trial

The Cervantes trial is a multicenter, randomized phase III study currently investigating whether radical surgery alone is non-inferior to the combination of radical surgery followed by adjuvant (chemo)radiotherapy in terms of disease-free survival in patients with intermediate-risk cervical cancer.

A total of 514 patients with early-stage (FIGO IB1–IIA) intermediate-risk cervical cancer are being randomized in a 1:1 ratio to one of two treatment arms. Arm A involves radical surgery without further adjuvant therapy, whereas Arm B includes external beam radiotherapy—optionally combined with brachytherapy and/or concomitant chemotherapy—after surgery.

Eligible patients must present with negative lymph nodes and at least one unfavorable prognostic feature, such as a tumor >4 cm, a tumor >2 cm with lymphovascular space invasion, deep stromal invasion of more than two-thirds, or a surgical margin of less than 3 mm [16]. Only HPV-associated squamous cell carcinoma and adenocarcinoma cases are included.

Patient outcomes will be monitored for three years after the last patient has been randomized for the analysis of the primary endpoint (disease-free survival), and for six years in total to assess overall survival. Results regarding the primary endpoint are anticipated by 2031 [16].

## 3. Locally Advanced Cervical Cancer (LACC)

Primary chemoradiation is recommended for patients with locally advanced cervical cancer (from stage IIB), as well as in cases of lymph node involvement, inoperability and the presence of preoperatively proven risk factors (L1, R1, G3 (questionable significance and only in combination with two other risk factors), neuroendocrine carcinoma, tumor > 4 cm) [3].

### 3.1. The EMBRACE Trials

Image guided brachytherapy is one of the most significant developments in the field of radiation therapy and especially in the treatment of LACC.

Initially the RetroEMBRACE study [17] and subsequently the EMBRACE I study [18] showed that MRI guided Brachytherapy results not only in excellent local control and pelvic control (92% and 87% at 5 years, respectively), but also a very good overall survival of 74% at 5 years.

The incidence of severe morbidity was limited per organ (grade ≥ 3, 3.2–8.5%; grade ≥ 4 0.5–3.0%), but considerable overall (grade ≥ 3, 18.4%; grade ≥ 4, 5.2%), especially for patients with stage III–IVA disease [18]. Very importantly, it was shown that by using interstitial implants with MRI guidance, tumours that were in the past deemed as untreatable (with extension to the pelvic wall or to the bladder/rectum wall) could be cured (OS at 5 years 64% in FIGO stage_2009_ IIIB and 52% in IVA).

The EMBRACE-I cohort study provides robust prospective clinical evidence for the feasibility and efficacy of MRI-guided image-guided adaptive brachytherapy (IGABT) in the management of locally advanced cervical cancer (LACC) [18]. It has also defined essential reference standards regarding individualized target volume delineation, optimal dosing strategies, and acceptable dose constraints for organs at risk. These findings serve as important benchmarks for both clinical practice and quality assurance in research settings. Based on this evidence, MRI-guided brachytherapy should be considered the standard of care in the treatment of LACC.

### 3.2. Uterus-11 Trial

The prospective randomized, multicenter Uterus-11 trial investigated whether patients with LACC stage IIB-IVA cervical cancer (FIGO 2009) benefit from surgical staging compared to clinical staging prior to planned primary platinum-based chemoradiation.

A total of 255 patients with locally advanced cervical cancer were randomized into two groups: 130 in the surgical arm and 125 in the clinical arm. Of these, 240 patients were included in the final analysis. Laparoscopic staging resulted in upstaging for 39 out of 120 patients (33%). Following a median follow-up of 90 months, no significant difference in disease-free survival (DFS) was identified between the groups (p = 0.084) [19]. Nonetheless, in patients with FIGO stage IIB cervical cancer, surgical staging proved to be superior to clinical staging regarding DFS (HR = 0.51, 95% CI: 0.30–0.86, p = 0.011). Moreover, a post hoc analysis showed that surgical staging was linked to enhanced cancer-specific survival (HR = 0.61, 95% CI: 0.40–0.93, p = 0.020) [19].

These results indicate that while no overall DFS benefit was observed between surgical and clinical staging in patients with locally advanced cervical cancer (LACC), surgical (laparoscopic) staging was associated with significantly better DFS and, as shown in post hoc analysis, improved cancer-specific survival in those with FIGO stage IIB disease [19].

The findings were supported by a recent large database analysis.

This study evaluated how surgical staging of para-aortic lymph nodes influences survival in patients with locally advanced cervical cancer receiving definitive chemoradiation. Among 3540 patients, surgical para-aortic staging was performed in 333 cases (9%). Positive lymph nodes were identified more frequently in those who underwent lymphadenectomy compared to those assessed only by imaging (27% vs. 13%, p < 0.001). Despite this, no significant difference in overall survival was observed between patients who had radiologic versus surgical para-aortic node evaluation (p = 0.80) [20]. The 4-year overall survival rates were similar between the groups, suggesting that para-aortic lymphadenectomy did not confer a survival advantage [20].

The international multicenter, randomized, prospective phase III study, PAROLA, aims to determine whether chemoradiation using a tailored external beam radiation field based on surgical staging and pathological examination of the para-aortic lymph node leads to improved 3-year disease-free survival compared to staging with PET/CT alone [21]. The results are expected to be presented in 2030.

### 3.3. OUTBACK Trial

The OUTBACK trial, a multicenter, randomized, and open-label study, aimed to determine whether the addition of adjuvant chemotherapy following standard chemoradiotherapy could enhance survival in patients with locally advanced cervical cancer. The study included patients with stage IB1 (FIGO 2008) and lymph node metastases, as well as those with stages IB2, II, IIIB, and IVA. Participants were randomly assigned in a 1:1 ratio to receive either standard cisplatin-based chemoradiotherapy alone (n = 456) or the same regimen followed by four additional cycles of adjuvant chemotherapy with carboplatin and paclitaxel (n = 463) [22].

The findings revealed no meaningful difference in survival outcomes between the two groups. The 5-year overall survival rate was 71% (95% CI: 66–75) in the group receiving chemoradiotherapy alone (116 deaths) and 72% (95% CI: 67–76) in the group receiving additional adjuvant chemotherapy (105 deaths) [22]. The absolute difference was 1% (95% CI: –6 to 7), with a hazard ratio of 0.90 (95% CI: 0.70–1.17; p = 0.81).

Adverse events were more common in the adjuvant chemotherapy group, with grade 3–4 neutropenia observed in 20% (71 patients) compared to 8% (34 patients) in the chemoradiotherapy-only group [22]. Similarly, anemia occurred in 18% (66 patients) versus 8% (34 patients), respectively. Serious adverse events affected 30% of patients receiving adjuvant chemotherapy and 22% of those treated with chemoradiotherapy alone, with infections being the most frequent complication.

Results from the OUTBACK trial indicated that administering adjuvant platinum-based chemotherapy following standard chemoradiation increased treatment-related toxicity without demonstrating a survival advantage. Consequently, this strategy is not recommended for routine use in unselected patients with locally advanced cervical cancer [22]. Similarly, in patients with high-risk features after radical hysterectomy, the addition of systemic chemotherapy post-chemoradiotherapy did not enhance disease-free or overall survival [23].

### 3.4. CALLA Trial: Evaluation of Durvalumab in Addition to Chemoradiation as First-Line Therapy for Locally Advanced Cervical Cancer

This phase III, multicenter, randomized, placebo-controlled trial evaluated the PD-L1 inhibitor durvalumab in combination with primary chemoradiotherapy (CRT) for selected LACC patients (Stage IB2-IIBN1 or ≥stage III, any N). The brachytherapy in this trial was 40% point A based. The 12-month progression-free survival (PFS) rate was 76.0% (71.3–80.0) with durvalumab and 73.3% (68.4–77.5) with placebo [24]. In this population of patients with LACC not selected for biomarkers, no relevant benefit was seen from adding durvalumab to CRT. No additional adverse events were observed with the addition of durvalumab to CRT [24].

The CALLA trial investigated the addition of durvalumab, a PD-L1 inhibitor, to standard chemoradiotherapy in a population not selected based on biomarker expression. The results did not show a significant enhancement in progression-free survival, suggesting that the integration of immune checkpoint inhibitors into frontline chemoradiotherapy requires further investigation and potentially biomarker-driven patient selection.

### 3.5. ENGOT-cx11 Trial/GOG-3047/KEYNOTE-A18: Evaluation of Pembrolizumab in Addition to Chemoradiation as First-Line Therapy for LACC

Pembrolizumab has shown encouraging results not only in palliative care but also, for the first time, in patients newly diagnosed with locally advanced cervical cancer (LACC).

In a multicenter, randomized, placebo-controlled phase III trial, participants received five cycles of either pembrolizumab (200 mg) or placebo administered every three weeks in combination with chemoradiotherapy. This was followed by 15 maintenance cycles of pembrolizumab (400 mg) or placebo every six weeks [25]. The patient population was similar to the one in the Calla trial, however in the current one, the node positivity was carefully defined for patients with stage IB2-IIB as 2 or more positive pelvic LN by MRI or CT or by PET-CT with SUV ≥ 2.5 or 1 or more positive PAN LN by MRI or CT or by PET-CT with SUV ≥ 2.5. In 90% of these patients, a volume prescribed brachytherapy was performed.

The median progression-free survival was not reached in either group; however, at 24 months, the progression-free survival rate was 68% in the CRT plus pembrolizumab group compared to 57% in the CRT plus placebo group [25]. The hazard ratio (HR) for disease progression or death was 0.70 (95% CI, 0.55–0.89; p = 0.0020) [25]. Overall survival at 24 months was 87% in the CRT plus pembrolizumab group and 81% in the CRT plus placebo group, with an HR for death of 0.73 (95% CI, 0.49–1.07) [25].

CRT, including volume-based brachytherapy, followed by pembrolizumab demonstrated a statistically significant and clinically meaningful improvement in progression-free survival, along with a favorable trend in overall survival, in patients with locally advanced high-risk cervical cancer. Additionally, the treatment exhibited a manageable safety profile.

Pembrolizumab was approved for use with chemoradiotherapy for treatment of patients with FIGO 2014 stage III–IVA cervical cancer by the FDA and it is anticipated that this trial will lead to the approval of pembrolizumab for the adjuvant treatment of patients with locally advanced cervical cancer undergoing primary CRT in Europe [25,26].

### 3.6. INTERLACE-Trial: Evaluation of Neoadjuvant Chemotherapy with Chemoradiation for Locally Advanced Cervical Cancer

The INTERLACE trial evaluated whether short-term weekly induction chemotherapy (IC) before standard chemoradiotherapy (CRT) enhances progression-free survival (PFS) and overall survival (OS).

This randomized, multicenter phase III study included 500 patients with FIGO stage IB1 (2008) with positive lymph nodes, IB2, IIA, IIB, IIIB, or IVA. Among them, 43% had pelvic lymph node involvement, while para-aortic node involvement was not permitted [27]. Brachytherapy in this trial was 70%-point A based. Patients were randomly assigned (1:1) to receive either CRT alone (5 cycles of weekly cisplatin) or IC (6 cycles of weekly carboplatin AUC2 and paclitaxel 80 mg/m^2^) followed by CRT at week 7 [27].

The 5-year progression-free survival (PFS) rate was 72% in the group receiving induction chemotherapy followed by chemoradiotherapy (IC/CRT), compared to 64% in the group treated with chemoradiotherapy (CRT) alone (HR = 0.65; 95% CI: 0.46–0.91; p = 0.013) [27]. Overall survival (OS) at five years was 80% in the IC/CRT group versus 72% in the CRT-only group (HR = 0.60; 95%CI: 0.40–0.91; p = 0.015). However, grade ≥ 3 adverse events were more frequent in the IC/CRT group (59%) compared to the CRT group (48%) [27].

These results suggest that incorporating induction chemotherapy prior to chemoradiotherapy offers significant improvements in both PFS and OS in patients with locally advanced cervical cancer [27]. However, potential limitations should be considered, including the long enrollment period and evolving standards of care in modern radiotherapy, such as image-guided brachytherapy [28].

## 4. Metastatic or Recurrent Cervical Cancer

The palliative treatment options for patients with recurrent or metastatic cervical cancer are limited and usually not very effective. In most cases, single-agent chemotherapies such as topoisomerase I inhibitors, gemcitabine or vinorelbine are administered, which not only offer little prognostic benefit but are also associated with significant side effects and reduced quality of life. The most important trials in this setting are depicted in Table 2.

### 4.1. KEYNOTE-826 Trial: Pembrolizumab in Addition to Taxane-Based Combination Chemotherapy +/− Bevacizumab for Primary Metastatic or Recurrent Cervical Cancer

The multicenter, randomized, placebo-controlled phase-III trial Keynote-826 investigated whether the addition of pembrolizumab leads to an extension of overall survival and progression-free survival in patients with persistent, recurrent or primary metastatic cervical cancer.

A total of 617 patients, either treatment-naïve or previously treated with radio(chemo)therapy, were included in the study. Participants received pembrolizumab (200 mg every three weeks) or a placebo alongside paclitaxel plus cisplatin or carboplatin, with or without bevacizumab.

Patients with PD-L1-positive cervical cancer experienced significantly longer progression-free survival (PFS) when pembrolizumab was added to standard therapy (10.5 months vs. 8.2 months, HR 0.58 [0.47–0.71], p < 0.001) [5]. Overall survival (OS) also improved significantly with pembrolizumab (24-month survival: 53.5% vs. 39.4%, HR 0.60 [0.49–0.74], p < 0.001) [5].

Notably, similar results were observed in the biomarker-unselected intention-to-treat population, which included a small subgroup of patients with PD-L1 <1 tumors [5]. In the overall study population, pembrolizumab significantly improved PFS (10.4 months vs. 8.2 months, HR 0.61 [0.50–0.74], p < 0.001) [5]. OS was also significantly extended (24-month survival: 52.1% vs. 38.7%, HR 0.63 [0.52–0.77], p < 0.001) when pembrolizumab was incorporated into standard therapy [5].

Subgroup analyses suggest that pembrolizumab’s efficacy is independent of bevacizumab administration. This indicates that patients ineligible for bevacizumab can still benefit from pembrolizumab. However, patients with PD-L1 <1 tumors did not appear to derive significant benefit from pembrolizumab, with an OS hazard ratio of 0.87 [0.50–1.52], though the wide 95% confidence interval overlapped with that of the overall population [5].

The incidence of grade ≥3 adverse events was 82.4% in the pembrolizumab plus chemotherapy group compared to 75.4% in the placebo plus chemotherapy group [5]. The most common grade 3–5 adverse events in both groups included anemia (30.3% vs. 26.9%), neutropenia (12.4% vs. 9.7%), and arterial hypertension (9.4% vs. 10.7%) [5]. Immune-related side effects typical of pembrolizumab were manageable in an outpatient setting and did not lead to any significant decline in quality of life.

The KEYNOTE-826 trial showed that combining pembrolizumab with platinum-based chemotherapy, with or without the addition of bevacizumab, significantly improved survival outcomes for patients with persistent, recurrent, or metastatic cervical cancer. Notably, patients whose tumors expressed PD-L1 experienced the most pronounced benefit, although improvements in progression-free and overall survival were observed across the broader patient population [5]. Consequently, for patients with primary metastatic or recurrent PD-L1-positive cervical cancer, the recommended standard treatment now includes a platinum-based chemotherapy regimen with or without bevacizumab and pembrolizumab. However, the effectiveness of this four-drug approach in patients with PD-L1 <1 tumors remains a subject of debate.

### 4.2. BEATcc: Atezolizumab in Addition to Platinum-Based Combination Chemotherapy + Bevacizumab for Primary Metastatic or Recurrent Cervical Cancer

The randomized, open-label phase-III BEATcc trial included 410 patients with persistent, recurrent or primary metastatic cervical cancer who had previously received no systemic therapy or only radio(chemo)therapy.

Patients were assigned in a 1:1 ratio to receive standard therapy (cisplatin or carboplatin, paclitaxel, and bevacizumab) with or without atezolizumab 1200mg.

Patients treated with atezolizumab experienced a median progression-free survival (PFS) of 13.7 months (95% CI: 12.3–16.6), compared to 10.4 months (95% CI: 9.7–11.7) in those receiving standard treatment, resulting in a hazard ratio (HR) of 0.62 (95% CI: 0.49–0.78; p < 0.0001) [29].

Interim analysis of overall survival (OS) showed a median OS of 32.1 months (95% CI: 25.3–36.8) in the atezolizumab group, versus 22.8 months (95% CI: 20.3–28.0) in the standard therapy group, with an HR of 0.68 (95% CI: 0.52–0.88; p = 0.0046) [29]. Grade ≥3 adverse events were reported in 79% of patients treated with the experimental regimen and in 75% of patients under standard treatment. Side effects such as diarrhea, arthralgia, pyrexia, and grade 1–2 rash were more common among patients receiving atezolizumab.

The BEATcc trial evaluated the efficacy of adding atezolizumab to a first-line regimen consisting of bevacizumab, platinum-based chemotherapy, and paclitaxel in patients with recurrent or metastatic cervical cancer. This combination led to statistically significant improvements in both progression-free and overall survival, highlighting it as a promising therapeutic option in this clinical setting [29].

### 4.3. ENGOT-cx6 + cx12 Trial: Tisotumab-Vedotin as a New Treatment Option for Cervical Cancer in the Recurrent Setting

Cervical cancer exhibits high tissue factor expression, which can be targeted using tisotumab vedotin (TV), an antibody-drug conjugate (ADC).

The ENGOT-cx6 trial is a multicenter, open-label, single-arm Phase II study designed to assess the clinical effectiveness of tisotumab-vedotin (2 mg/kg i.v. q3w) in 101 patients with recurrent cervical cancer [30]. These patients had previously undergone dual chemotherapy with bevacizumab and had received up to two prior systemic therapies. The median progression-free survival (PFS) was 4.2 months (3.0–4.4), while the median overall survival (OS) was 12.1 months (9.6–13.9). After a median follow-up of 10 months, the trial reported a clinical response rate of 24% (95% CI, 16–33), including a 7% complete remission rate [30].

The most frequently reported treatment-related side effects included alopecia (38%), nosebleeds (30%), nausea (27%), conjunctivitis (26%), fatigue (26%), and dry eyes (23%). Serious adverse events of grade 3 or higher were recorded in 28% of patients, with the most common being neutropenia (3%), fatigue (2%), and ulcerative keratitis (2%). Additionally, 2% of patients experienced various forms of peripheral neuropathy, including sensory, motor, sensorimotor, and general peripheral neuropathy. One patient death due to septic shock was deemed treatment-related by the investigator. Tisotumab vedotin, an antibody–drug conjugate targeting tissue factor, demonstrated durable antitumor responses with an acceptable safety profile in patients with previously treated recurrent or metastatic cervical cancer. These findings from the ENGOT-cx6 study support its consideration as a treatment option in this context [30].

The phase-III ENGOT-Cx12 trial investigated whether TV has a favourable effect on survival compared to chemotherapy (topotecan, vinorelbine, gemcitabine, irinotecan or pemetrexed) in patients with recurrent or metastatic cervical cancer as a second or third-line therapy [31]. A total of 502 patients were randomized, of which 253 were assigned to the tisotumab-vedotin group and 249 to the chemotherapy group.

The median overall survival was significantly longer in the tisotumab-vedotin group compared to the chemotherapy group, at 11.5 months (95% confidence interval [CI], 9.8–14.9) versus 9.5 months (95% CI, 7.9–10.7) [31]. This translated to a 30% reduction in the risk of death with tisotumab-vedotin compared to chemotherapy (hazard ratio, 0.70; 95% CI, 0.54–0.89; two-sided p = 0.004). Median progression-free survival was 4.2 months (95% CI, 4.0–4.4) with tisotumab-vedotin and 2.9 months (95% CI, 2.6–3.1) with chemotherapy (hazard ratio, 0.67; 95% CI, 0.54–0.82; two-sided p < 0.001) [31]. The confirmed objective response rate was 17.8% in the tisotumab-vedotin group, compared to 5.2% in the chemotherapy group (odds ratio, 4.0; 95% CI, 2.1–7.6; two-sided p < 0.001). In patients with recurrent cervical cancer, tisotumab-vedotin was significantly more effective than chemotherapy [31].

### 4.4. ENGOT-cx8 Trial

The open-label, multicenter phase Ib/II ENGOT-cx8 trial investigated the toxicity and dose escalation arms of tisotumab-vedotin in combination with bevacizumab, pembrolizumab or carboplatin in recurrent or metastatic cervical cancer.

The recommended phase II dosage for tisotumab vedotin in combination with bevacizumab (Arm A), pembrolizumab (Arm B), or carboplatin (Arm C) was successfully established [32]. In the dose-escalation cohorts, the antitumor efficacy and safety of tisotumab vedotin at this recommended dose were further evaluated in combination with carboplatin as first-line therapy (Arm D), and with pembrolizumab as both first-line (Arm E) and second- or third-line therapy (Arm F) [32].

The primary endpoint for this phase was the objective response rate (ORR), with a total enrollment of 142 patients [32]. Since no dose-limiting toxicities were observed, the recommended dose for phase II was set at tisotumab vedotin 2 mg/kg in combination with either bevacizumab 15 mg/kg, pembrolizumab 200 mg, or carboplatin AUC 5—administered every three weeks [32].

The ORR was 54% for Arm D (first-line with carboplatin), 41% for Arm E (first-line with pembrolizumab), and 35% for Arm F (second-/third-line with pembrolizumab) [32]. The median duration of response was 8.6 months for Arm D, not reached for Arm E, and 14.1 months for Arm F.

Grade 3 or higher adverse events occurring in 15% or more of patients included anemia, diarrhea, nausea, and thrombocytopenia in Arm D, while only anemia reached this threshold in Arm F [32]. No grade ≥3 adverse events of ≥15% incidence were reported in Arm E.

Overall, the study demonstrated that combining tisotumab vedotin with bevacizumab, pembrolizumab, or carboplatin resulted in manageable toxicity and showed promising antitumor activity in patients with recurrent or metastatic cervical cancer [32].

## 5. Upcoming Treatment Options—The Most Promising ADCs

ADCs were developed to release cytotoxic substances into the tumor cell in a targeted manner by means of antibody–antigen interaction. This should not only increase the effectiveness of the therapy, but also reduce drug-related adverse events. The most promising ADCs for recurrent and metastatic cervical cancer are presented in this chapter.

### 5.1. Trastuzumab Deruxtecan (Targeting HER2)

The antibody-drug conjugate Trastuzumab deruxtecan (T-DXd) is already approved for other tumor entities, such as HER2-expressing breast cancer. The target is human epidermal growth factor 2 (HER2), which is also expressed in cervical cancer.

In the multicenter, open-label phase II study “DESTINY-PanTumor02”, the efficacy and safety of T-DXd was investigated in various tumor entities that express HER2 (immunohistochemistry 3+/2+) and were locally advanced or metastatic.

A total of 267 patients from seven tumor groups, including 40 patients with cervical cancer, received T-DXd 5.4 mg/kg once every 3 weeks.

The median follow-up time was 13 months. The overall response rate (ORR) across all patients was 37% (n = 99; 95%CI, 31.3–43.2), with responses observed in all groups. The median progression-free survival (PFS) across the overall study population was 6.9 months (95% CI: 5.6–8.0), and the median overall survival (OS) was 13.4 months (95% CI: 11.9–15.5) [33].

In the subgroup of patients with centrally confirmed HER2 IHC 3+ expression (n = 75), the objective response rate (ORR) reached 61% (95% CI: 49.4–72.4) [33]. The median duration of response (DOR) was 22.1 months (95% CI: 9.6 to not reached). This group also showed a median PFS of 11.9 months (95% CI: 8.2–13.0) and a median OS of 21.1 months (95% CI: 15.3–29.6) [33].

Investigator-assessed ORRs for all patients by cohort were 50.0% for cervical cancer (95% CI, 33.8–66.2). In the subgroup with centrally confirmed HER2 IHC 3+ expression (n = 75), the investigator-assessed ORR for cervical cancer was 75.0% (n = 8; 95% CI, 34.9–96.8) [33].

Grade ≥3 drug-related adverse events occurred in 40.8% of patients, with 10.5% experiencing drug-related interstitial lung disease (ILD), leading to three deaths [33].

In conclusion, the most significant benefit was observed in the IHC 3+ population. T-DXd represents a promising therapeutic approach for patients with HER2-positive cervical cancer under strict risk-benefit assessment and close monitoring during therapy [33].

### 5.2. Sacituzumab Govitecan, Sacituzumab Tirumotecan (Targeting TROP2)

TROP-2 is another potential target for ADCs, as it is highly expressed in nearly 90% of cervical cancers.

Sacituzumab govitecan is an antibody-drug conjugate (ADC) composed of a humanized anti-TROP-2 antibody linked to SN-38, the active metabolite of irinotecan, via a pH-sensitive, hydrolyzable linker designed to enable bystander killing in the tumor microenvironment [34]. In 2023, the FDA granted approval for sacituzumab govitecan in the treatment of patients with unresectable, locally advanced, or metastatic hormone receptor-positive, HER2-negative breast cancer who had previously received at least two prior systemic therapies, including endocrine treatment [34]. The multicenter, single-arm, phase-II clinical trial EVER-132-003 is investigating the efficacy and safety of Sacituzumab govitecan in patients with solid tumours, including patients with advanced cervical cancer after treatment failure of platinum- and paclitaxel-based chemotherapy.

Sacituzumab govitecan 10 mg/kg i.v. was administered on days 1 and 8 of 21 days cycle.

Interim analysis data revealed strong anti-tumor activity, with a 50% objective response rate (ORR) and durable treatment benefits, along with a notable advantage in patients previously treated with immunotherapy [34].

Sacituzumab tirumotecan (SKB264/MK-2870) utilizes the same antibody as Sacituzumab govitecan but features a linker engineered for greater stability. Its novel topoisomerase I inhibitor is a derivative of belotecan, exhibiting comparable in vitro activity to both belotecan and the payload used in Sacituzumab govitecan.

The following phase II study evaluated the efficacy and safety of Sacituzumab tirumotecan in patients with recurrent or metastatic cervical cancer who had a progressive disease during or after platinum doublet chemotherapy and had received no more than 2 systemic therapies. 3 or 5 mg/kg Q2W + pembrolizumab 400 mg Q6W was given [35].

The ORR was 57.9% (22/38, 3 unconfirmed), with 3 complete remissions. The median DoR was not reached and the 6-month DoR rate was 82.1% [35].

A total of 47.4% experienced grade ≥ 3 treatment-related adverse events, which in most cases led to dose reduction.

Sacituzumab tirumotecan plus pembrolizumab showed promising and durable antitumor activity with a manageable safety profile [35].

The open-label, randomized, multicenter phase-III study ENGOT-cx20 is currently recruiting patients, who receive either a monotherapy with Sacituzumab tirumotecan or a treatment of physician’s choice as second line treatment for recurrent or metastatic cervical cancer.

## 6. The Most Promising Current Clinical Trials

There are currently several promising ENGOT clinical trials underway, the results of which are expected in the coming years and are likely to refine the treatment of cervical cancer. The following chapter provides a brief overview of future research directions.

As mentioned earlier, the results of the PAROLA and ENGOT-Cx16 studies will provide far-reaching insights into the further treatment of cervical cancer.

The ENGOT-cx19/GEICO/eVOLVE trial is a phase III, randomized, double-blind, placebo-controlled, multicentre, global study which explores the efficacy and safety of Volrustomig, a bispecific antibody that blocks PD-1 and CTLA-4, in women with high-risk LACC (FIGO 2018 stage IIIA to IVA cervical cancer) who have not progressed following platinum-based CRT [36]. Women with LACC will be randomized in a 1:1 ratio to receive treatment with Volrustomig or Placebo [36].

The Senticol III trial is a large prospective multicenter international randomized study designed to validate the Sentinel Lymph Node mapping technique in early cervical cancer [36]. This “validation study” will compare the outcome of patients with negative SLN (experimental arm) vs. patients with negative SLN + Pelvic Lymph Node dissection (reference arm) [36].

## 7. Conclusions

With respect to surgical treatment, a constant de-escalation in local management as well as lymph node management can be observed. With respect to systemic therapy, checkpoint-inhibitors have already taken an important place alone, in combination with chemotherapy, and in combination with chemoradiation in the various settings of cervical cancer. Moreover, the first antibody-drug conjugates demonstrate efficacy in recurrent cervical cancer.

In patients with early-stage cervical cancer and particular low-risk features (lesions of ≤2 cm with limited stromal invasion), SH is non-inferior to radical hysterectomy in terms of 3-year incidence of pelvic recurrence. Furthermore, sentinel lymphadenectomy is steadily taking a more prominent role and replacing systematic pelvic lymphadenectomy in patients with low-risk cervical cancer. With respect to postoperative adjuvant therapy, studies are in planning to identify improved treatment options for patients with intermediate-risk cervical cancer.

Advancements in the systemic treatment of cervical cancer have expanded therapeutic options beyond standard chemoradiotherapy. The integration of immune checkpoint inhibitors and antibody–drug conjugates offers new avenues, particularly in advanced and recurrent settings. However, personalized treatment selection based on biomarkers such as PD-L1 remains essential to optimize outcomes. Ongoing trials will further refine the role of these agents across different stages of disease.

The combination of platinum-based chemotherapy and pembrolizumab, with or without bevacizumab, has emerged as a new standard for treating primary metastatic or recurrent PD-L1 positive cervical cancer. Furthermore, checkpoint inhibitors (CPIs) are expected to be utilized in additional clinical settings, supported by several positive randomized clinical trials. Notably, approvals for these expanded indications are still pending but anticipated soon.

Regarding locally advanced cervical cancer (LACC), the past two decades have seen significant advancements in image-guided radiotherapy, particularly with the introduction of MRI-guided brachytherapy. On the systemic treatment front, two escalation strategies have been investigated alongside chemoradiation: neoadjuvant chemotherapy and adjuvant CPI therapy. However, as the full publication on neoadjuvant chemotherapy is not yet available, its precise role in clinical practice remains to be determined.

Adjuvant pembrolizumab in LACC has been approved by FDA and approval in Europe is awaited. Furthermore, antibody-drug conjugates like tisotumab vedotin may offer a promising therapeutic approach for the future management of cervical cancer.

## Figures and Tables

**Table 1:** Overview of new surgical treatment options for cervical cancer.

Trial	SHAPE	LESSER	ConCerv	LACC	Senticol
Design	multicenter	multicenter	multicenter	multicenter	multicenter
Population	low-risk CC	IA2-IB1	low-risk CC	early-stage CC	early-stage CC
N patients	700	40	100	631	259
Treatment	radical HE vs. simple HE	simple HE vs. mod. radical HE	conservative surgery	minimally invasive surgery vs. open abdominal radical HE	SLNB vs. PL
Follow-up	recurrence after 3 years 2.17% vs. 2.52%	3-year DFS 95% vs. 100%	recurrence after 2 years 3.5%	3-year DFS 91% vs. 97%	DFS 85.1% vs. 80.4%
OS		5-year OS 90% vs. 91%	-	3-year OS 93.8% vs. 99%	
Safety		postop. complications 15% vs. 25%	-	-	

**Table 2:** Overview of new systemic treatment options for recurrent or metastatic cervical cancer.

Trial	KEYNOTE-826	BEATcc	ENGOT-cx6	ENGOT-cx8
Phase	3	3	2	1b/2
Design	multicenter	multicenter	multicenter	-
Population	rec or met CC	rec or met CC	rec CC	rec or met CC
N patients	617	410	101	142
Treatment	pembrolizumab	atezolizumab	tisotumab-vedotin	tisotumab-vedotin
ORR %	-	-	-	54.5%
PFS	mPFS 10.4 mo	mPFS 13.7 mo	mPFS 4.2 mo	-
OS	24-mo OS 52.1%	mOS 32.1 mo	mOS 12.1 mo	-
Safety	serious AEs 82.4%	serious AEs 79%	serious AEs 28%	serious AEs 15%

## Data Availability

No new data were created or analyzed in this review.

## Abbreviations

LACC	locally advanced cervical cancer
PD-L1	programmed cell death ligand 1
CPIs	immune checkpoint inhibitors
OS	overall survival
FIGO	International Federation of Gynecology and Obstetrics
HR	hazard ratio
PFS	progression-free survival
DFS	Disease-free survival
DSS	disease-specific survival
SH	simple hysterectomy
SLNB	sentinel lymph node biopsy
SLN	sentinel lymph node
MAC	macrometastases
MIC	micrometastases
MIS	minimally invasive surgery
ITC	isolated tumour cells
PL	pelvic lymphadenectomy
PLND	pelvic lymph node dissection
RT	radiotherapy
TV	Tisotumab-vedotin
